# Human delta like 1-expressing human mesenchymal stromal cells promote human T cell development and antigen-specific response in humanized NOD/SCID/IL-2R$\upgamma$^null^ (NSG) mice

**DOI:** 10.1038/s41598-021-90110-z

**Published:** 2021-05-19

**Authors:** Do Hee Kwon, Jae Berm Park, Joo Sang Lee, Sung Joo Kim, Bongkum Choi, Ki-Young Lee

**Affiliations:** 1 Department of Immunology and Samsung Biomedical Research Institute, Sungkyunkwan University School of Medicine, 2066 Seobu-ro, Jangan-gu, Suwon, 16419 Gyeonggi-do Korea; 2 Department of Surgery, Samsung Medical Center, Sungkyunkwan University School of Medicine, Seoul, Republic of Korea; 3 Department of Health Sciences and Technology, Samsung Advanced Institute for Health Sciences and Technology, Samsung Medical Center, Sungkyunkwan University, Seoul, Republic of Korea; 4 Department of Precision Medicine, Sungkyunkwan University School of Medicine, Suwon-Si, 440-746 Kyonggi-Do Korea; 5 Single Cell Network Research Center, Sungkyunkwan University School of Medicine, Suwon, 16419 Republic of Korea; 6 GenNBio, Inc., Seoul, Republic of Korea; 7 Department of Medicine, Sungkyunkwan University School of Medicine, Suwon-Si, Kyonggi-Do, 440-746 Korea

**Keywords:** Immunology, Stem cells

## Abstract

Human delta-like 1 (hDlk1) is known to be able to regulate cell fate decisions during hematopoiesis. Mesenchymal stromal cells (MSCs) are known to exhibit potent immunomodulatory roles in a variety of diseases. Herein, we investigated in vivo functions of hDlk1^-^hMSCs and hDlk1^+^hMSCs in T cell development and T cell response to viral infection in humanized NOD/SCID/IL-2Rγ^null^ (NSG) mice. Co-injection of hDlk1^-^hMSC with hCD34^+^ cord blood (CB) cells into the liver of NSG mice markedly suppressed the development of human T cells. In contrast, co-injection of hDlk1^+^hMSC with hCD34^+^ CB cells into the liver of NSG dramatically promoted the development of human T cells. Human T cells developed in humanized NSG mice represent markedly diverse, functionally active, TCR V$\upbeta$ usages, and the restriction to human MHC molecules. Upon challenge with Epstein-Barr virus (EBV), EBV-specific hCD8^+^ T cells in humanized NSG mice were effectively mounted with phenotypically activated T cells presented as hCD45^+^hCD3^+^hCD8^+^hCD45RO^+^hHLA-DR^+^ T cells, suggesting that antigen-specific T cell response was induced in the humanized NSG mice. Taken together, our data suggest that the hDlk1-expressing MSCs can effectively promote the development of human T cells and immune response to exogenous antigen in humanized NSG mice. Thus, the humanized NSG model might have potential advantages for the development of therapeutics targeting infectious diseases in the future.

## Introduction

Humanized mice are valuable pre-clinical tools for developing new therapeutics and for studying human immune responses to infections by pathogens, development of human immune cells, and so on^1–10^. More than 20 years after the first successful engraftment of human leukocytes and hematopoietic organs into mice^11^, many different humanized mice have been reported and used for a variety of purposes^1–11^. Recent advances in humanized mice have been focusing on methods for generating humanized mice with at least two purposes: (1) to make it simple to generate humanized mice; and (2) to make it efficacious to guarantee successful reconstitution of human immune cells without any graft-versus-host disease (GVHD) when grafting human cells in mice. As part of these efforts, many laboratories have developed new methodologies to develop humanized mice, including human fetal thymic and liver tissues (SCID-hu thy-liv mice)-engrafted humanized mice^5^ and intravenous injected humanized mice^12^. We have recently reported that intrahepatic injection of CD34^+^ human (h) cord blood (CB) cells to conditioned newborn mice can successfully induce reconstitution of human immune cells in NOD/SCID/IL-2Rγ^null^ (NSG) mice^8^. Compared to the generation of SCID-hu thy-liv mice-engrafted humanized mice, generating humanized mice by intrahepatic injection is simple so that they could be applied to solve current scientific issues including the development of therapeutics targeting human diseases and academic interests related to cellular and molecular functions of immune responses.

Mesenchymal stromal cells (MSCs) have multi-potent effects and functions in immunomodulatory responses^13–16^. Co-transplantation of ex vivo-expanded human MSCs with hematopoietic stem cells can hasten hematopoietic recovery followed by bone marrow transplantation in animal models and humans^16–22^. In addition, MSCs have shown immuno-suppressive effects on T cells^23^. The production of HLA-G5 by MSCs can suppress T-cell proliferation and cytotoxicity induced by natural killer and T cells^24,25^. Moreover, cell-to-cell contact between MSCs and activated T cells can induce IL-10 production essential to stimulate the release of soluble HLA-G5, resulting in systemic immunosuppressive effects on tumor growth^26,27^. Nevertheless, whether MSCs might affect T cell development and generation in an in vivo system remains unclear. T cell development and generation in vivo are affected by a variety of environmental systems including cells, soluble mediators, and receptor-ligand interactions^14,28–30^. Among them, Notch signaling plays a key role for the decision of cell fate during the developmental stage. It also functionally contributes to the commitment of T cell lineage^31–33^. In mammals, four different notch receptors (NOTCH1, NOTCH2, NOTCH3, and NOTCH4), have been identified. They can transduce intracellular signaling related to cell proliferation and differentiation^31^. Recent studies have shown that delta-like 1 homolog (Dlk1) can directly interact with NOTCH1 receptor and modulate cell fate determination, terminal differentiation, and proliferation^34–36^. However, whether Dlk1 is functionally associated with T cell development, especially in an in vivo system, has not been reported yet.

In this study, we tried to answer the following two questions: (1) Could Dlk1 regulate human T cell development in humanized mice? and (2) Could MSC-induced immunomodulatory effects, especially in T cell development and function, be regulated by Dlk1? To answer these questions, we utilized humanized NSG mice generated by intrahepatic injection with hCD34^+^ CB cells alone or together with hMSC or hMSC expressing Dlk1. We then assessed the development of human T cells. Additionally, we examined whether antigen-specific T cell response was mounted in humanized mice challenged with Epstein-Barr virus (EBV). Our data demonstrated that co-injection of hDlk1-expressing MSCs dramatically promoted the development of human T cells and that human T cells exhibited functionally active, diverse TCR V$\upbeta$ usages with active response to human MHC molecules. Upon challenge with EBV, EBV-specific hCD8^+^ T cells were effectively generated. These results suggest that Dlk1-expressing MSCs can functionally facilitate human T cell development in humanized NSG mice. The humanized mouse model developed in this study together with diverse humanized mouse model available could be used to study various immune diseases and viral diseases in the future.

## Materials and methods

All experiments involving animals were approved by the Laboratory Animal Research Center (LARC) of the Samsung Biomedical Research Institute in Seoul, Korea and adhered to the ARVO Statement for the Use of Animals in Ophthalmic and Vision Research and the ARRIVE (Animal Research: Reporting of In Vivo Experiments) guidelines. The human protocol for the experiments with human materials was approved by the Institutional Review Boards of Samsung Medical Center (SMC) (IRB No.: 2010-09-060).

### Mice and human fetal liver tissue and ethical statement

6- to 8-week-old NOD/SCID/IL-2Rγ^null^ (NSG) mice were purchased from the Jackson Laboratory (Bar Harbor, ME, USA). All mice were bred as a homozygous line and maintained under specific-pathogen-free (SPF) conditions in accordance with ethical guidelines for the care of mice at the Laboratory Animal Research Center (LARC) of the Samsung Biomedical Research Institute (SBRI) and the Samsung Medical Center (SMC) in Seoul, Korea. The animal protocols (No. 20160617001) were approved by the Institutional Animal Care and Use Committee (IACUC) of the SBRI and the SMC. Human fetal liver tissues of gestational age 17–22 weeks were obtained from the SMC. Informed, written consent was acquired from the patients or legal guardian. The human protocol for the experiments with human materials was approved by the Institutional Review Boards of SMC (IRB No.: 2010–09-060).

### Isolation of human fetal liver-derived mesenchymal stromal cells (hFL-MSCs)

Human fetal liver tissues were obtained from SMC at 17–22 weeks of gestation. Single cell suspensions of fetal liver tissues were made by previously reported methods^37^. Mononuclear cells (MNCs) were isolated from single cells of fetal liver tissues using Ficoll-Hypaque density gradient centrifugation and then washed with culture medium. Cells were seeded into 75 cm^2^ flasks at a density of 5 × 10^5^ cells per cm^2^ in alpha modified Eagle’s medium ($\alpha$-MEM, Hyclone Lab, Inc, Logan, Utah, USA) supplemented with 10% FBS and 1X antibiotic–antimycotic solution (Hyclone Lab). After 3 to 5 days, non-adherent cells were removed by washing and then culture flasks were refilled with culture medium. At approximately 80% confluence, cells were detached with 0.25% trypsin and 1 mM EDTA solution (Hyclone Lab), plated into 75 cm^2^ culture flasks at a density of 5 × 10^5^ cells per cm^2^, and cultured at 37 °C under 5% CO_2_ atmosphere.

### Construction of human delta-like 1 (Dlk1) retroviral vector

Human Dlk1 cDNA was synthesized from total RNA extracted from AFT024 cells expressing human Dlk1. cDNA synthesized was performed using 1 μg of purified total RNA in a reaction volume of 20 μl containing 250 mM Tri-HCl, pH 8.3, 375 mM KCl, 15 mM MgCl_2_, 0.1 M DTT, 10 mM each dNTP, 20 units of RNase inhibitor, 0.5 μg/μl oligo(dT)12–18 primer, and 200 units of SuperScript reverse transcriptase (Invitrogen, CA, USA). RT reaction was performed at 37 °C for 1 h. Synthesized cDNA samples were purified using a QIAquick PCR Purification Kit (Qiagen GmbH, Hilden, Germany) and used as a template for PCR. PCR primers for hDlk1 (accession number: MN003836) were specifically designed as follows: forward, 5′-GGGTCCATGACCGCGACCGAAGCC-3′; reverse, 5′-CCTAGGTTAGATCTCCTCGTCGCC-3′. All amplicons were cloned into BamHI and AvrII sites of a pLXRN retroviral vector (Clontech Laboratories, Inc., CA, USA). The pLXRN vector and the purified hDlk1 DNA were linearized with BamHI and AvrII to prepare compatible ends for ligation using T4 DNA ligase (New England Biolabs, Pickering, ON, USA).

### Transfection and production of retrovirus

Retroviruses were prepared in GP2-293 cells, an HEK 293-based packing cell line. Transfection was performed in serum-free OptiMEM I (Invitrogen) with lipofectamine 2000 (Invitrogen). pLXRN-Dlk1 was transfected into GP2-293 cells with pVSV-G being, according to the manufacturer’s instructions. At 8–10 h after transfection, culture medium was aspirated and then complete medium was added followed by incubation at 37 °C for an additional 48–72 h under 5% CO_2_ atmosphere. Supernatant containing viruses was filtered through a 0.22 μm Steriflip filter (Millipore, MA, USA) through centrifugation at 50,000 g for 90 min at 4 °C. After removing the supernatant, viruses were resuspended in 1% of the original volume in TNE (50 mM Tris–HCl, pH 7.8, 130 mM NaCl, and 1 mM EDTA) buffer and incubated at 4 °C overnight.

### Infecting human fetal liver-derived mesenchymal stromal cells (hFL-MSCs) with retroviruses

Human fetal liver-derived MSCs were seeded into 75 cm^2^ flasks and cultured in culture medium (α-MEM supplemented 10% FBS, 100 U/ml penicillin, and 100 μg/ml streptomycin) until 80% confluence. MSCs were incubated with viruses at a multiplicity of infection (MOI) of 10 or 15 in MSCs culture medium containing 8 μg/ml polybrene. Infected cells were selected with 600 μg/ml neomycin G418 (Invitrogen) in culture medium at 37 °C under 5% CO_2_ for two weeks. At two weeks after selection, culture medium was freshly replaced. Cells were grown in culture medium, consisting of alpha modified Eagle’s medium (a-MEM, Hyclone Lab, Inc, Logan, Utah, USA) supplemented with 10% FBS and 1X antibiotic–antimycotic solution (Hyclone Lab) in a humidified atmosphere of 5% CO_2_ at 37 °C. At 80% confluence, cells were detached and seeded at a density of 2 × 10^5^ cells per cm2 in T75 flask (Nunc, Denmark) and used up to five passages for the experiments.

### Immunofluorescence staining

MSCs and Dlk1-expressing MSCs were plated on chamber slides (LabTek II; Nalge Nunc International, Rochester, NY, USA), cultured for 2 days at 37 °C, fixed with 4% paraformaldehyde, permeabilized with ice-cold conditioned ethanol and acetic acid buffer, and further incubated at −20 °C for 10 min. After the cells were washed with 1X PBS containing 0.1% Triton X-100, the cells were then incubated with 5ug/ml anti-DLK1 antibody (abcam, Cambridge, UK) in 1X PBS containing 1% BSA and 0.1% Tween-20 at 4 °C overnight. Decant the primary antibody mixture solution and wash the cells three times in 1X PBS, and subsequently incubated cells with the FITC-conjugated goat anti-mouse IgG H&L secondary antibody (abcam, Cambridge, UK) used at a 1:1000 dilution in 1X PBS containing 1% BSA and 0.1% Tween-20. the cells were incubated in the dark for 1 h at room temperature. After washing, nuclear DNA was stained using 4, 6-diamidino-2-phenylindole (DAPI) as counterstain and the slides were mounted. The stained slides were observed using an Olympus BX51 fluorescence microscope (Olympus, Japan) with 10X/22 numeric aperture and 40$\times$/0.75 numeric aperture objective, and photographs were taken by a microscope digital camera DP50 (Olympus, Japan) and image-pro plus 5.1 software.

### Isolation of human CD34^+^ cells from umbilical cord blood

Human CB samples were acquired from normal full-term deliveries after obtaining informed parental consent according to guidelines established by Samsung Medical Center, Seoul, Korea. Mononuclear cells (MNCs) were isolated using Ficoll-Hypaque density gradient centrifugation. hCD34^+^ cells were purified by previously reported methods^6–9^. The purity of isolated cells was estimated by flow cytometric analysis with antibodies specific for anti-hLin-fluorescein isothiocyanate (FITC) conjugate (Becton Dickinson, NJ, USA) and anti-hCD34-phycoerythrin (PE) conjugate (BD Pharmingen™, CA, USA). hLin^-^hCD34^+^ cells (> 95%) were used for the generation of humanized NSG mice.

### Humanized mice generated by intrahepatic co-injection with hCD34^+^ CB cells and Dlk1-expressing MSCs or MSCs into conditioned neonatal NSG mice

Busulfan (Busulfex, Ben Benue Laboratories, Inc., Bedford, OH, USA) was dissolved in dimethyl sulfoxide (DMSO, Sigma Chemical Co., St. Louis, MO, USA), diluted in 0.9% saline, and then injected intravenously into newborn NSG mice via the facial vein at a dose of 15 mg/kg body weight in a total volume of 50 μl^8^. At 8 to 24 h after injection, isolated hCD34^+^ cells and Dlk1-expressing MSCs or MSCs were co-injected into the liver of conditioned newborn NSG mice at a concentration of 2 × 10^5^ cells of CB cells and 1 × 10^6^ cells of Dlk1-expressing MSCs or MSCs.

### Flow cytometric analysis

Peripheral blood mononuclear cells (PBMCs) were collected from tail veins of humanized NSG mice at 8, 12, 16, and 20 weeks after transplant. To remove red blood cells (RBCs), cells were treated with 1X RBC Lysis Buffer (Invitrogen) according to the manufacturer's instructions. Single-cell suspensions were prepared, and mononuclear cells (MNCs) were isolated by previously reported methods^6–9^. Isolated MNCs were stained with hCD45- allophycocyanin (APC) conjugated, or -peridinin chlorophyll protein (PerCP)-Cy5.5 conjugate, hCD3-PerCP)-Cy5.5 conjugated, hCD8-, hHLA-DR-, and h$\mathrm{\gamma \delta }$TCR-phycoerythrin (PE) conjugated, h $\alpha \beta$ TCR-, hCD19-fluorescein isothiocyanate (FITC) conjugated, or hCD45RO-PE-Cyanine 7 (Cy7) conjugated antibody. To characterize Dlk1-expressing MSCs and MSCs, hCD34-, hCD45-, hCD44-, hCD90-FITC, hCD73-, hCD105-, hHLA-DR-PE, and hCD14-APC conjugated antibodies were used. They were purchased from Invitrogen. Cells were stained with appropriate antibodies in 100 μl PBS containing 0.2% BSA and 0.05% sodium azide for 15 min on ice. Flow cytometry analysis was performed using a FACSAria (BD Biosciences). Ten thousand to 1,000,000 events were acquired per sample and analyzed using FACSDiva (BD Biosciences) software and the data analyzes using FlowJo (BD Biosciences) software.

### Immunohistochemistry

All of the humanized NSG mice spleen tissues were harvested, fixed in 10% formalin and embedded in paraffin. Immunohistochemistry was performed on 4 μm tissue sections fixed on slide glass, deparaffinized with xylene and rehydrated with ethanol. Immunohistochemistry (IHC) was performed using Dako Real Envision Detection System, Peroxidase/DAB Rabbit/Mouse (DAKO, Glostrup, Denmark) kit according to the manufacturer’s instructions. Primary antibody used for IHC was rabbit polyclonal anti-human CD3 (1:1000, DAKO, Glostrup, Denmark). Stained slides were observed using an Olympus CX41 light microscope (Olympus, Japan) with 10X/22 numeric aperture and 40x/0.75 numeric aperture objective, and photographs were taken by a microscope digital camera DP50 (Olympus, Japan) and image-pro plus 5.1 Software. All samples were counterstained with hematoxylin and eosin (H&E).

### Mixed lymphocytes reaction (MLR) assay

Human CD3^+^ lymphocytes as responder cells were isolated from spleens of humanized NSG mice using either a MACS human CD3 MicroBead Kit (Miltenyi Biotec, GlodBach, Germany) or an autoMACS™ Cell Separator (Miltenyi Biotec) according to the manufacturer's instructions. MLR assay was performed by previously reported methods^8^. Briefly, stimulator cells were prepared from PBMCs of healthy human volunteers (*n* = 3) and irradiated at 30 Gy. Then 2 × 10^5^ hCD3^+^ responder cells purified from the humanized mice were plated in triplicate into a 96-well U-bottom plate and incubated without or with irradiated 4 × 10^5^ stimulator cells. After 3 days, MTT assay was performed to check cell proliferation according to the manufacture's instruction (Roche Diagnostics, Indianapolis, IN).

### Human cytokine release assay

Human CD3^+^ lymphocytes were purified from humanized NSG mice as mentioned above. Human cytokine release assay was performed by previously reported methods^8^. Briefly, 1 × 10^6^ hCD3^+^ cells derived humanized NSG mice were plated in triplicate into 24-well plates and co-cultured in the presence or absence of irradiated PBMCs (2 × 10^6^ cells) isolated from healthy human volunteers (*n* = 3). After 3 days, supernatants were harvested and levels of human cytokines such as hIL-2 and hIFN-γ were measured using ELISA Ready-SET-go kit and according to the manufacture's instruction (Invitrogen).

### Human TCR Vβ repertoire analysis

Human TCR Vβ repertories were analyzed with a TCR Vβ repertoire kit (Beckman Coulter/Immunotech, France), as previously reported methods^8^. Samples were analyzed using a FACSAria (BD Biosciences). Data were analyzed using FACSDiva and GraphPad Prism software (GraphPad Software, La Jolla, CA, USA).

### Experimental EBV infection into humanized mice

To produce EBV, B95-8 (Marmoset B-lymphoblastoid cell line) was used as described in the previous report^38^. Humanized mice were generated using hCD34^+^ CB cells alone (*n* = 10) or together with hDlk1-expressing hFL-MSCs (*n* = 10) as described above. At 20 weeks after transplantation, humanized mice were challenged with 100 μl EBV concentrate (2 × 10^6^ EBV copy or equivalent to approximately 1.5 × 10^3^ TD_50_ of B95.8 EBV virus solution.) was injected via intravenous injection. At 4 weeks after infection, peripheral blood samples were isolated from each group of humanized mice (*n* = 5 in each group). Other mice (*n* = 5 in each group) were sacrificed and spleen cells were isolated.

### EBV-specific pentamer staining

EBV-specific HLA-A*0201 pentamer (EBV LMP-1, YLLEMLWRL) was purchased from ProImmune Ltd (Oxford, UK). Peripheral blood and spleen were isolated from EBV-challenged humanized mice. To remove red blood cells (RBCs), cells were treated with 1X RBC Lysis Buffer (Invitrogen) according to the manufacturer's instructions. Single-cell suspensions were prepared from peripheral blood and spleen using standard procedures. These cells were stained with anti-hCD45-APC, anti-hCD3-PerCP-Cy5.5, anti-hCD8-PE, and EBV-specific HLA-A*0201 pentamer was labeled with FITC in 100 μl PBS containing 0.2% BSA and 0.05% sodium azide for 30 min on ice. Flow cytometry analysis was performed on a FACSAria (BD Biosciences). Ten thousand to 1,000,000 events were acquired per sample and analyzed using a FACSDiva (BD Biosciences) or a FlowJo (BD Biosciences) software.

### Statistical analysis

Data are presented as mean values ± S.D. as indicated and analyzed using Student’s two-tailed *t*-test. The *p*-value was considered significant at values less than 0.05, statistically significant (*p < 0.05, **p < 0.01, ***p < 0.001, ****p < 0.0001).

## Results

### Experimental design and research issues

**Figure 1:**
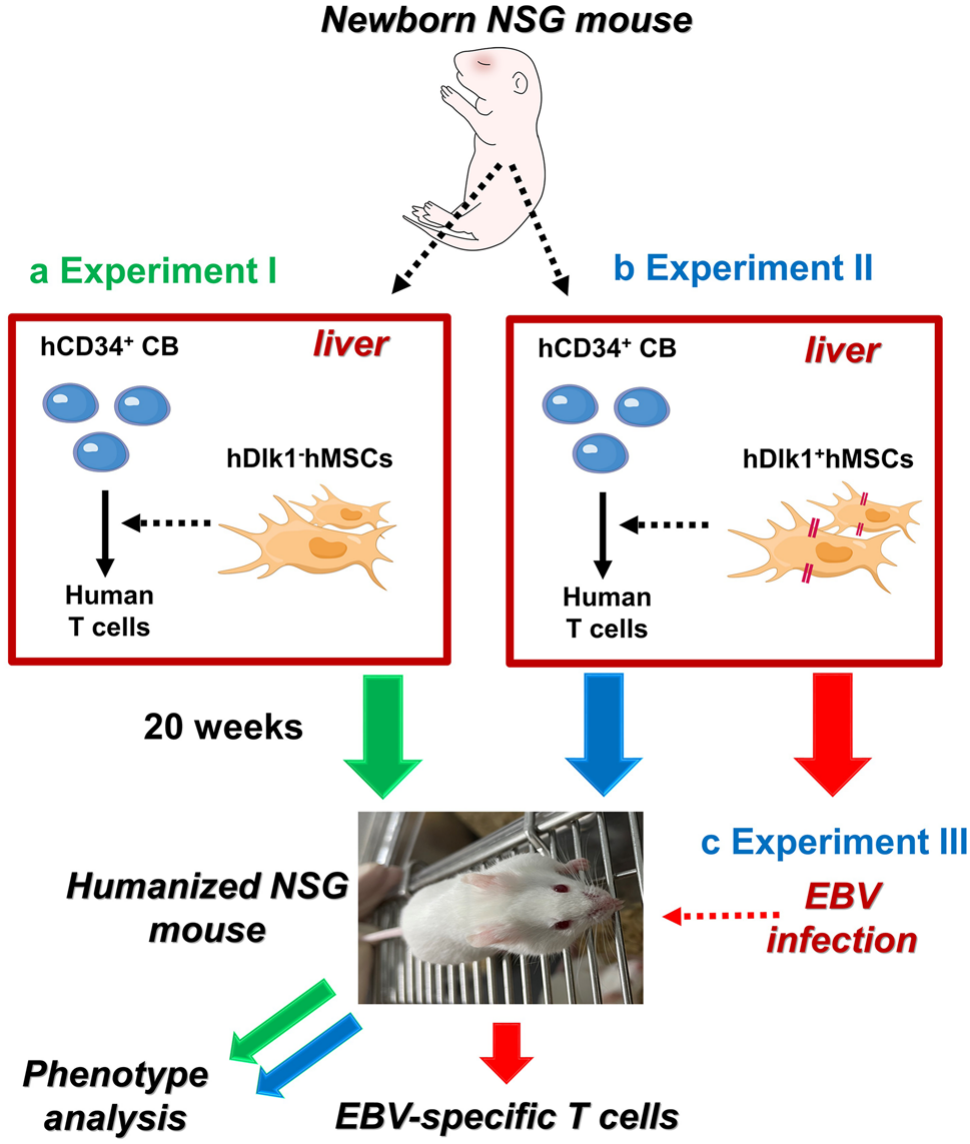
Experimental designs for the generation of humanized NSG mice in this study. Three sets of humanized NSG mice were designed and generated, represented as Experiment I (**a,** green lines), Experiment II (**b,** blue lines), and Experiment III (**c,** red lines) as described in Materials and Methods. (**a**) Experiment I: hCD34^+^ CB cells or hCD34^+^ CB cells plus hMSCs (hDlk^-^) cells were injected into livers of newborn NSG mice. (**b**) Experiment II: hCD34^+^ CB cells or hCD34^+^ CB cells plus hDlk^+^hMSCs cells were injected into livers of newborn NSG mice. Reconstitution of human T and B cells was assessed in humanized NSG mice as described in the main text. (**c**) Experiment III: Humanized NSG mice were generated with hCD34^+^ CB cells or hCD34^+^ CB cells plus hDlk^+^hMSCs cells. After 20 weeks, EBV was used to challenge humanized NSG mice as described in Materials and Methods.

In this study, we had two fundamental questions to be possibly addressed. Although previous reports have demonstrated immunomodulatory effects of mesenchymal stromal cells (MSCs) on T cells^13–16,24,25^, in vivo functions of MSCs related to T cells are experimentally insufficient. Therefore, we first asked whether MSCs could affect T cell development using humanized NSG mice co-injected with or without MSCs (Fig. 1a, Experiment I). If so, is it possible to regulate MSC-induced T cell development using delta-like 1 (Dlk1) molecule (Fig. 1b, Experiment II)? It is known that Notch-mediated signaling plays a key role in T cell development^31–33^. Additionally, Dlk1 putatively can interact with Notch1 receptor, thereby regulating cellular development^34–36^. To address the second issue, we generated humanized NSG mice (Fig. 1b, Experiment II). To address the issues mentioned above, we performed an in vivo EBV-challenged experiment to see whether EBV-specific T cells could be mounted in the humanized mice (Fig. 1c, Experiment III).

### Human fetal liver-derived mesenchymal stromal cells (hFL-MSCs) can suppress the development of human T cells in NOD/SCID/IL-2R$\gamma$^null^ (NSG) mice generated by intrahepatic co-injection with hCD34^+^ cord blood (CB) cells

**Figure 2:**
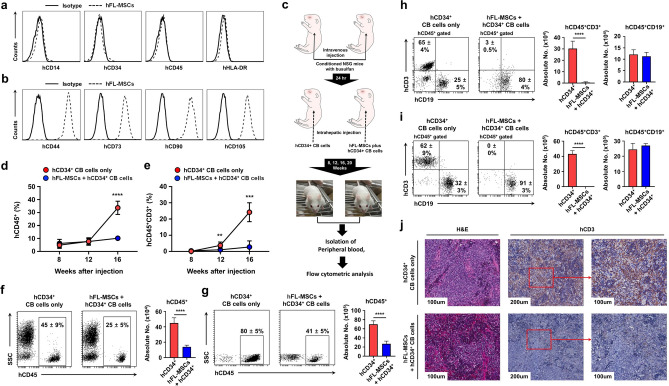
Human T cell development is suppressed in humanized NSG mice generated with hCD34^+^ CB cells together with hFL-MSCs. (**a **and** b**) Human MSCs were prepared from fetal liver tissues as described in Materials and methods. These cells were stained with anti-CD14, anti-CD34, anti-CD45, anti-HLA-DR, anti-CD44, anti-CD73, anti-CD90, and anti-CD105 antibodies followed by flow cytometry analysis. Isotype antibody in each sample was used as control. (**c**) Schematic diagram showing the generation of humanized NSG mice. Busulfan was intravenously injected into NSG newborn mice (*n* = 30). After 24 h, hCD34^+^ CB cells alone (*n* = 13) or together with hFL-MSC cells (*n* = 13) were co-injected into livers of conditioned newborn NSG mice as described in Materials and methods. (**d **and** e**) Peripheral blood samples were isolated from humanized NSG mice generated by using hCD34^+^ CB cells alone (*n* = 5) or together with MSCs (*n* = 5) at different time points as indicated. These cells were then stained with anti-hCD45 and anti-hCD3 antibody followed by flow cytometry analysis on a FACSAria. Ten thousand to 1,000,000 events were acquired per sample and hCD45^+^ (**d**) and hCD45^+^hCD3^+^ (**e**) were analyzed using FACSDiva software. Percentages of cells were obtained by manual flow cytometric gating method. Data are shown as the average of five different mice in each group (± S.D). *, *p* < 0.05; ***, *p* < 0.001; ****, *p* < 0.0001. (**f–i**) Peripheral blood (**f **and** h)** and spleen **(g **and** i)** tissues samples were isolated from humanized NSG mice generated with hCD34^+^ CB cells alone (*n* = 8) or together with hFL-MSCs (*n* = 8) at end time point (20 weeks). Cells were stained with anti-hCD45, anti-hCD3, and anti-hCD19 antibodies as described in Materials and methods. Percentages and absolute numbers of cells were obtained by manual flow cytometric gating and counted. Data are presented as the average of eight different mice in each group (± S.D). ****, *p* < 0.0001. (**j**) Spleens were isolated from humanized NSG mice, fixed with 10% formalin, and embedded in paraffin. Paraffin embedded tissue sections were stained with hematoxylin and eosin (H&E) and anti-hCD3 antibody as described in Materials and Methods. Stained slides were observed using an Olympus BX40 light microscope. Photographs were taken with a microscope digital camera DP50 and analyzed with an Image-pro plus 5.1 software.

To explore the functional role of human MSCs (hMSCs) in the generation of human T cells in humanized mice, hMSCs were isolated from human fetal liver (hFL) as described in Materials and Methods. These cells were then incubated with antibodies specific for hCD14, hCD34, hCD45, hHLA-DR, hCD44, hCD73, hCD90, and hCD105 molecules. As shown in Fig. 2a,b, flow cytometric analysis revealed that these isolated MSCs were negative for hematopoietic or endothelial cell markers such as hCD14, hCD34, hCD45, and hHLA-DR (Fig. 2a), whereas they were significantly positive for MSC markers such as hCD44, hCD73, hCD90, and hCD105 (Fig. 2b) as compared with those of isotype control. The identity of MSC was consistent with that shown in previous reports^39,40^. By using isolated hMSCs and hCD34^+^ cord blood (CB) stem cells, humanized NOD/SCID/IL-2R$\gamma$^null^ (NSG) mice were generated (Fig. 2c). Briefly, conditioned NSG newborn mice pre-treated with busulfan were intra-hepatically injected with hCD34^+^ CB stem cells alone or together with hMSC cells as depicted in Fig. 2c. To see the development of human T cells in humanized NSG mice, peripheral blood samples were isolated from tail veins at different times as indicated in Fig. 2d,e. Cells were stained with antibodies specific for hCD45, hCD3, and hCD19 molecules. hCD45^+^ and hCD45^+^hCD3^+^ T cells were gradually increased in humanized NSG mice injected with hCD34^+^ CB cells alone in a time dependent manner compared to those in humanized NSG mice injected with human fetal liver-MSCs (hFL-MSCs) plus hCD34^+^ CB cells (Figs. 2d,e, red circles *vs.* blue circles; Supplementary Fig. S1, hCD45^+^ cells; Supplementary Fig. S2, hCD45^+^hCD3^+^ T cells), whereas marginal increases in hCD45^+^hCD19^+^ cells could be seen in humanized NSG mice injected with hFL-MSCs plus hCD34^+^ CB stem cells (Supplementary Fig. S3, red circles *vs.* blue circles), supposing that hMSCs might suppress the generation of human T cells in humanized NSG mice established by intrahepatic injection^8^.

To verify the above results in more detail, humanized mice were sacrificed at 20 weeks after they were engrafted. Peripheral bloods and spleens were isolated from humanized mice. The reconstitution of human T and B cells was then evaluated using a flow cytometric analysis with antibodies to hCD45, hCD3, and hCD19 molecules. Similar to Fig. 2d, hCD45^+^ cells were markedly increased in peripheral blood (Fig. 2f, 45 ± 9% *vs.* 25 ± 5%; red bar *vs.* blue bar) and spleen (Fig. 2g, 80 ± 5% *vs.* 41 ± 5%; red bar *vs.* blue bar) of humanized NSG mice generated with hCD34^+^ CB cells alone than those of humanized mice generated with hCD34^+^ CB cells together with hFL-MSCs. Consistent with Fig. 2e, marked increases of hCD45^+^hCD3^+^ cells could be observed in humanized NSG mice generated with hCD34^+^ CB cells alone than in humanized mice generated with hCD34^+^ CB cells together with hFL-MSCs (Fig. 2h, peripheral blood, 65 ± 4% *vs.* 3 ± 0.5%, red bar *vs.* blue bar; Fig. 2i, spleen, 62 ± 9% *vs.* 0 ± 0%, red bar *vs.* blue bar). However, marginal differences in hCD45^+^hCD19^+^ cells could be detected in both humanized NSG mice (hCD45^+^hCD19^+^ cells in Fig. 2h, peripheral blood and Fig. 2i, spleen). Additionally, significant increase in hCD3^+^ cells in the spleen was confirmed using immunohistochemistry analysis (Fig. 2j, hCD34^+^ CB cells alone *vs.* hFL-MSCs + hCD34^+^ CB). These results suggest that hFL-MSCs may induce the suppression of human T cell development in humanized NSG mice co-injected with hCD34^+^ CB cells.

### hDlk1-expressing MSCs cells promote the development of human T cells in humanized NSG mice

**Figure 3:**
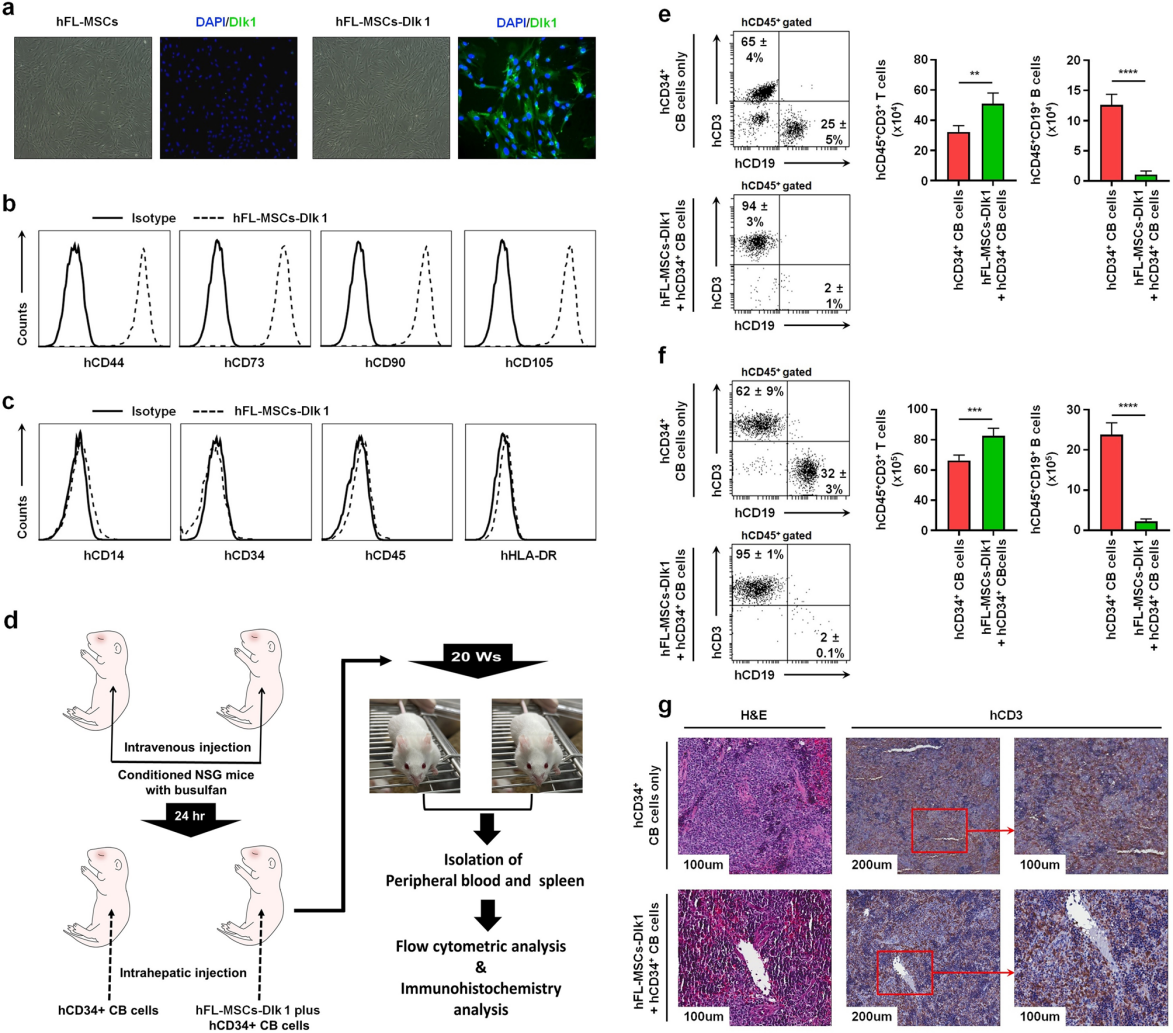
Generation of humanized NSG mice engrafted with hCD34^+^ CB cells together with human delta-like 1 (hDlk1)-expressing MSCs. (**a**) Human FL- MSCs were incubated with viruses at a multiplicity of infection (MOI) of 10 or 15 in hFL-MSCs culture medium containing 8 μg/ml polybrene. These transduced cells were selected for two weeks as described in Materials and Methods. Immunofluorescence staining was performed for hFL-MSC or hDlk1-expressing hFL-MSC cells as described in Materials and Methods. Stained slides were observed using an Olympus BX51 fluorescence microscope. Photographs were taken with a microscope digital camera DP50 and analyzed using an Image-pro plus 5.1 software. (**b** and **c**) hDlk1-expressing hFL-MSC cells were stained with anti-CD14, anti-CD34, anti-CD45, anti-HLA-DR, anti-CD44, anti-CD73, anti-CD90, and anti-CD105 antibodies followed by flow cytometry analysis. Isotype antibody in each sample was used as control. (**d**) Schematic diagram showing the generation of humanized NSG mice. Busulfan was intravenously injected into NSG newborn mice (*n* = 30). After 24 h, hCD34^+^ CB cells alone (*n* = 15) or together with hFL-MSC-hDlk1 cells (*n* = 15) were co-injected into livers of conditioned newborn NSG mice as described in Materials and method. (**e** and **f**) Peripheral blood (**e**) and spleen (**f**) tissues samples were isolated from humanized NSG mice generated with hCD34^+^ CB cells alone (*n* = 8) or together with hFL-MSCs-hDlk (*n* = 8). Cells were stained with anti-hCD45, anti-hCD3, and anti-hCD19 antibodies as described in Materials and methods. Percentages and absolute numbers of cells were obtained by manual flow cytometric gating and counted. Data are presented as average values of eight different mice in each group (± S.D). **, *p* < 0.01; ***, *p* < 0.001; ****, *p* < 0.0001. (**g**) Spleens were isolated from humanized NSG mice, fixed in 10% formalin, and embedded in paraffin. Paraffin embedded tissue sections were stained for hematoxylin and eosin (H&E) and anti-hCD3 antibody as described in Materials and Methods. Stained slides were observed using an Olympus CX41 light microscope. Photographs were taken with a microscope digital camera DP50 and analyzed with an Image-pro plus 5.1 software.

We have previously reported that notch signaling can facilitate the maintenance of self-renewal of hCD34^+^ CB cells in vitro and that it can induce effective reconstitution of human T cells in vivo humanized mice^6^. The putative interaction between Dlk1 and notch 1 in vitro and in vivo can regulate normal tissue development^34–36^. It is known that Notch signaling plays a key role for initial commitment to the T cell lineage, thereby regulating subsequent steps of T cell development^31–33^. Therefore, we asked whether hDlk1-mediated Notch signaling could compensate the suppressive effect of hMSCs in T cell development. To explore this issue, hDlk1-expressing hFL-MSCs (hFL-MSCs-Dlk1) cells were generated. The hDlk1 gene was then cloned and inserted into retroviral vector (Supplementary Fig. S4) as described in Materials and Methods. hDlk1-contained retroviruses were stably transduced into isolated hFL-MSCs as described in Material and Methods. hDlk1 expression was then confirmed by immunofluorescence microscopy (Fig. 3a, hFL-MSCs *vs.* hFL-MSCs-Dlk1). Next, to rule out the possibility that the expression of hDlk1 could affect characteristics of MSCs, hFL-MSCs-Dlk1 cells were characterized using antibodies as described in Figs. 2a and 2b. Consistently, hDlk1-expressed MSCs showed strong expression of MSC markers such as hCD44, hCD73, hCD90, and hCD105 (Fig. 3b), but not hematopoietic or endothelial cell markers such as hCD14, hCD34, hCD45, or hHLA-DR (Fig. 3c).

We then further generated humanized NSG mice injected with hCD34^+^ CB cells alone or hCD34^+^ CB cells plus hFL-MSCs-Dlk1 cells as depicted in Fig. 3d. At 20 weeks after engrafting cells, cells were isolated from peripheral bloods and spleens of humanized NSG mice and subjected to flow cytometric analysis to assess the development of human T and B cells. Interestingly, hCD45^+^hCD3^+^ cells were significantly increased in the peripheral blood of humanized NSG mice generated with hFL-MSCs-Dlk1 plus hCD34^+^ CB cells than in the peripheral blood of humanized mice generated with hCD34^+^ CB cells alone (Fig. 3e, 94 ± 3% *vs.* 65 ± 4%; absolute number of hCD45^+^hCD3^+^ cells, closed green bar *vs.* closed red bar). A similar increase of hCD45^+^hCD3^+^ cells could be detected in spleens of humanized NSG mice generated with hFL-MSCs-Dlk1 plus hCD34^+^ CB cells (Fig. 3f, 95 ± 1% *vs.* 62 ± 9%; absolute number of hCD45^+^hCD3^+^ cells, closed green bar *vs.* closed red bar). Such significant increase was also confirmed by immunohistochemistry analysis of spleen (Fig. 3g, hCD3 in hFL-MSCs-Dlk1 + hCD34^+^ CB *vs.* hCD34^+^ CB cells alone). However, marked attenuation of hCD45^+^hCD19^+^ cells were observed in humanized mice generated with hFL-MSCs-Dlk1 plus hCD34^+^ CB cells than in humanized mice generated with hCD34^+^ CB cells alone (Fig. 3e,f, hCD45^+^hCD19^+^ cells; absolute number of hCD45^+^hCD19^+^ cells, closed green bar *vs.* closed red bar). These results suggest that hDlk1-expressing hFL-MSCs may facilitate the development of human T cells, whereas they might suppress the development of human B cells in humanized NSG mice generated with hFL-MSCs-Dlk1 plus hCD34^+^ CB cells.

### Human T cells developed in humanized NSG mice co-injected with hCD34^+^ CB cells plus hFL-MSCs-Dlk1 cells show driver T cell repertoires and immune-competitive cells restricted to human MHC

**Figure 4:**
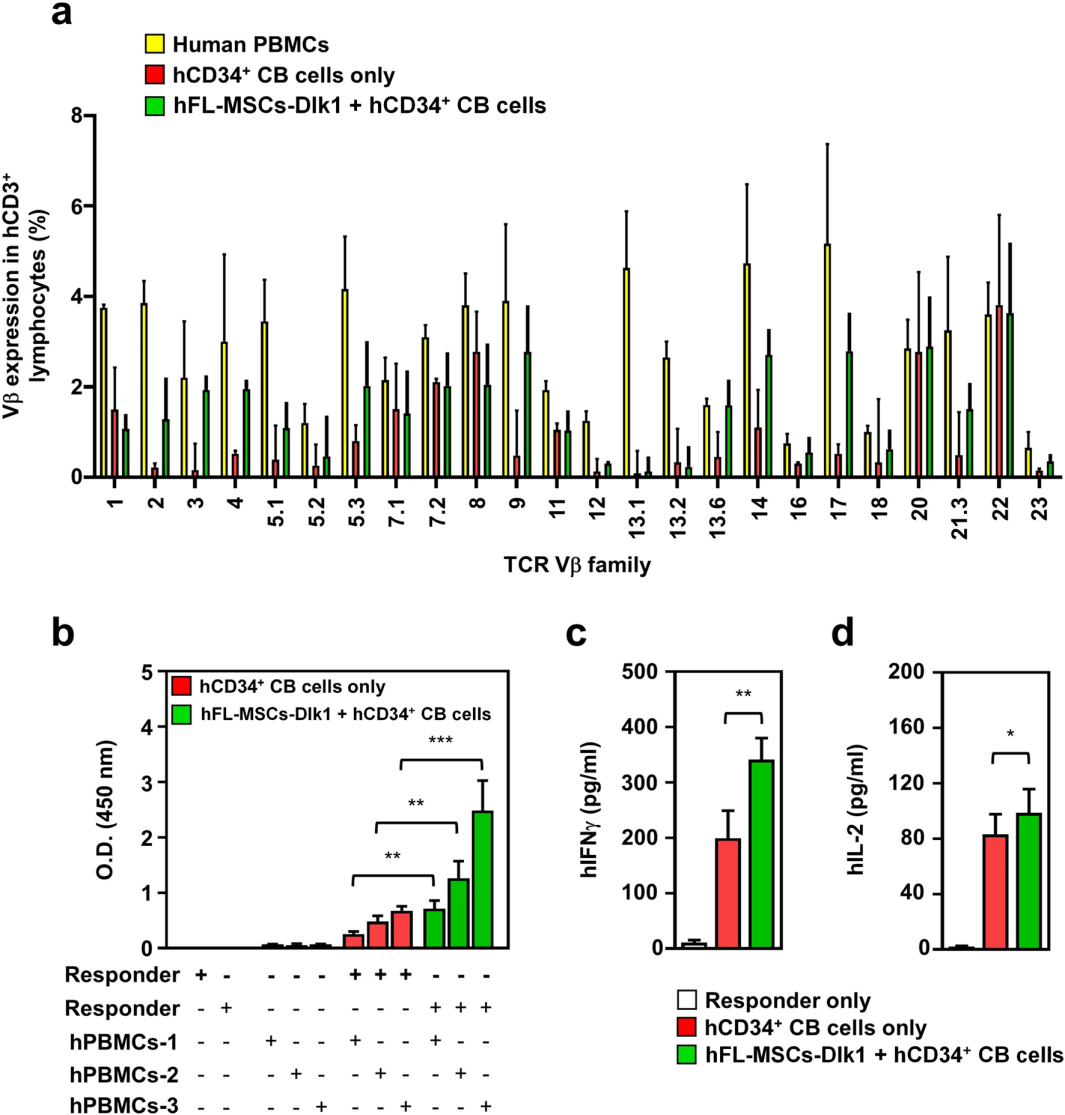
Characterization of humanized mice generated with hDlk1-expressing hFL-MSCs and hCD34^+^ CB cells. (**a**) Peripheral blood samples were isolated from humanized mice generated with hCD34^+^ CB cells alone (*n* = 5) or together with hDlk1-expresing hFL-MSC cells (*n* = 5) as described in Fig. 3d. Single cells were prepared as described in Materials and Methods. These cells were stained with a TCR Vβ repertoire kit according to the manufacturer's instructions. PBMCs were also isolated from normal healthy volunteers (*n* = 3) and stained with the TCR Vβ repertoire kit. Samples were analyzed using a FACSAria. Data are presented as the average of triplicate samples (± S.D). (**b**) Human CD3^+^ lymphocytes as responder cells were isolated from spleens of humanized NSG mice and then used for MLR assay as described in Materials and methods. Data are presented as the average of triplicate samples (± S.D). **, *p* < 0.01; ***, *p* < 0.001. (**c** and **d**) Purified human CD3^+^ T cells were co-cultured in the presence or absence of irradiated PBMCs isolated from healthy human volunteers as described in Materials and methods. After 3 days, supernatants were harvested and levels of human cytokines such as hIFN-γ (**c**) and hIL-2 (**d**) were measured with ELISA Kits according to the manufacture's instruction. Data are presented as the average of triplicate samples (± S.D). *, *p* < 0.05; **, *p* < 0.01.

Since the development of human T cells markedly appeared in humanized NSG mice generated with hFL-MSCs-Dlk1 plus hCD34^+^ CB cells, we assessed whether these T cells could be drivers in terms of TCR repertoire and whether they could functionally recognize human MHC molecules. In order to do that, PBMCs were isolated from humanized NSG mice at 20 weeks after engraftment. Their diversities were then compared with those of normal human PBMCs. When cells were stained with antibodies to twenty-four Vβ-T cell usages, human T cells derived from humanized NSG mice were significantly diverse, similar to the diversity of normal human periphery (Fig. 4a: yellow square, normal human PBMCs; red square, PBMCs of hCD34^+^CB cells only; green square, PBMCs of hDlk1-expressed MSCs plus hCD34^+^ CB cells), indicating T cells developed in humanized NSG mice had a diverse repertoire of Vβ-T cells receptors. Next, we asked whether human T cells could functionally recognize human MHC molecules. In order to do that, we performed mixed lymphocyte reaction (MLR) assay. Human CD3^+^ T cells as responder cells were purified from spleens of both humanized NSG mice. Stimulator cells were prepared from allogenic PBMCs of human healthy volunteers. Human CD3^+^ T cells derived from humanized NSG mice were co-cultured with different responder cells for 3 days. PMBCs derived from both humanized NSG mice showed significant proliferation in the presence of allogenic human PBMCs (Fig. 4b, hPBMCs-1, hPBMCs-2, and hPBMCs-3). Interestingly, the proliferative ability was much higher for T cells derived from humanized NSG mice generated with hDlk1-expressed MSCs plus hCD34^+^ CB cells than in T cells derived from humanized NSG mice generated with hCD34^+^ CB cells alone (Fig. 4b, closed green bars *vs.* closed red bars). When human cytokines such as hIFN-γ and hIL-2 were measured in cultures after MLR reaction, levels of hIFN-γ and hIL-2 were significantly higher in T cells derived from humanized NSG mice generated with hDlk1-expressed MSCs plus hCD34^+^ CB cells (Fig. 4c, hIFN-γ; Fig. 4d, hIL-2). These results suggest that human T cells derived from humanized NSG mice generated by hDlk1-expressed MSCs plus hCD34^+^ CB cells are restricted to human MHC molecules.

### EBV-specific T cells are effectively generated in humanized NSG mice obtained using hDlk1-expressing MSCs plus hCD34^+^ CB cells

**Figure 5:**
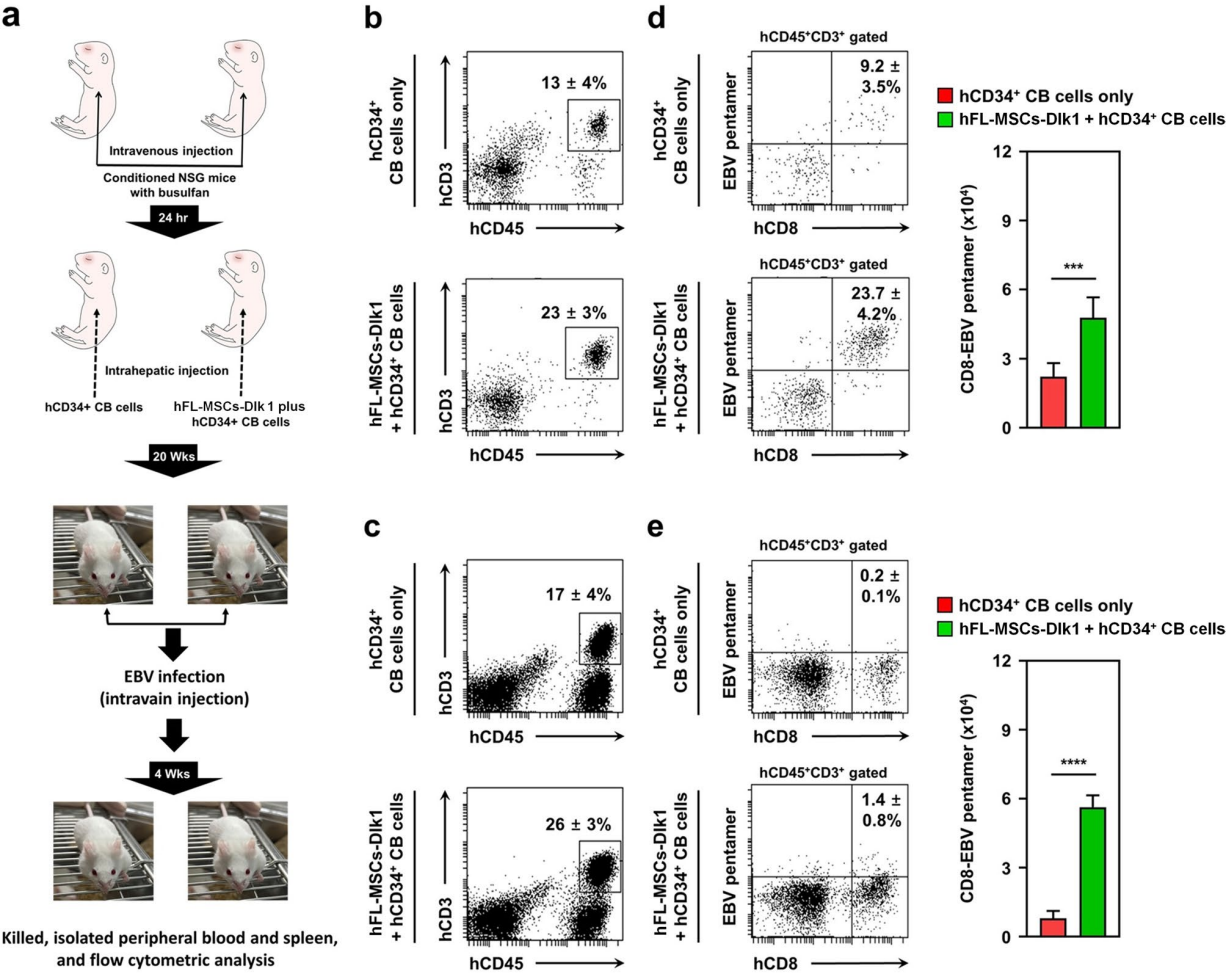
EBV infection induces the generation of EBV-specific T cells in humanized NSG mice generated with hDlk1-expressing hFL-MSCs. (**a**) A protocol for performing EBV infection. Humanized NSG mice were generated with hCD34^+^ CB cells alone (*n* = 10) or together with hDlk1-expressing hFL-MSCs (*n* = 10) as described in Materials and methods. After 20 weeks, EBVs were injected to humanized mice via intravenous infection as described in Materials and Methods. (**b** and **c**) At 4 weeks after EBV infection, peripheral blood (**b**) and spleen tissue (**c**) samples were isolated from humanized mice generated with hCD34^+^ CB cells alone (*n* = 5) or together with hDlk1-expressing hFL-MSCs (*n* = 5). Cells were prepared as described in Materials and Methods and stained with anti-hCD45 and hCD3 antibodies. Percentages of cells were obtained by manual flow cytometric gating method. Data are presented as the average of triplicate samples (± S.D). (**d** and **e**) PBMCs (**d,** *n* = 5) and spleen (**e,** *n* = 5) were isolated from humanized NSG mice. Cells stained with anti-hCD45, anti-hCD3, anti-hCD8, and anti-EBV pentamer as described in Materials and methods. Percentages and absolute numbers of cells were obtained by manual flow cytometric gating and counted. Data are presented as the average of triplicate samples (± S.D). ***, *p* < 0.001; ****, *p* < 0.0001.

Having shown the above results, we finally examined whether antigen-specific human T cell response could be effectively mounted in humanized NSG mice. In order to do that, 1.5 × 10^3^ TD_50_ of a B95-8 strain of EBV was inoculated into humanized NSG mice (Fig. 5a) as described in Materials and Methods. At four weeks after inoculation, mice were sacrificed and human T cell responses in peripheral blood and spleen to challenged EBV were characterized. hCD45^+^hCD3^+^ cells were significantly higher in humanized NSG mice generated by hDlk1-expressing MSCs plus hCD34^+^ CB cells than that those in humanized NSG mice generated using hCD34^+^ CB cells alone (Fig. 5b, peripheral blood: 23 ± 3% *vs.* 13 ± 4%; Fig. 5c, spleen: 26 ± 3% *vs.* 17 ± 4%). To assess antigen-specific T cells against EBV, we evaluated EBV-specific hCD8^+^ T cells in humanized NSG mice. EBV pentamer staining results revealed that hCD45^+^hCD3^+^hCD8^+^EBV pentamer^+^ cells were significantly higher in peripheral blood and spleen of humanized NSG mice generated using hDlk1-expressed MSCs plus hCD34^+^ CB cells than those of humanized NSG mice generated using hCD34^+^ CB cells alone (Fig. 5d, peripheral blood: 23.7 ± 4.2% *vs.* 9.2 ± 3.5%; Fig. 5e, spleen: 1.4 ± 0.8% *vs.* 0.2 ± 0.1%). Consistently, absolute EBV-specific hCD8^+^ T cell numbers were elevated in the peripheral blood and spleen of humanized NSG mice generated by hDlk1-expressed MSCs plus hCD34^+^ CB cells (Fig. 5d, peripheral blood, closed red bar *vs.* closed green bar; Fig. 5e, spleen, closed red bar *vs.* closed green bar). These results suggest that EBV-specific hCD8^+^ T cells are efficiently mounted in humanized mice generated by hDlk1-expressing MSCs plus hCD34^+^ CB cells than those in humanized mice generated by using hCD34^+^ CB cells.

**Figure 6:**
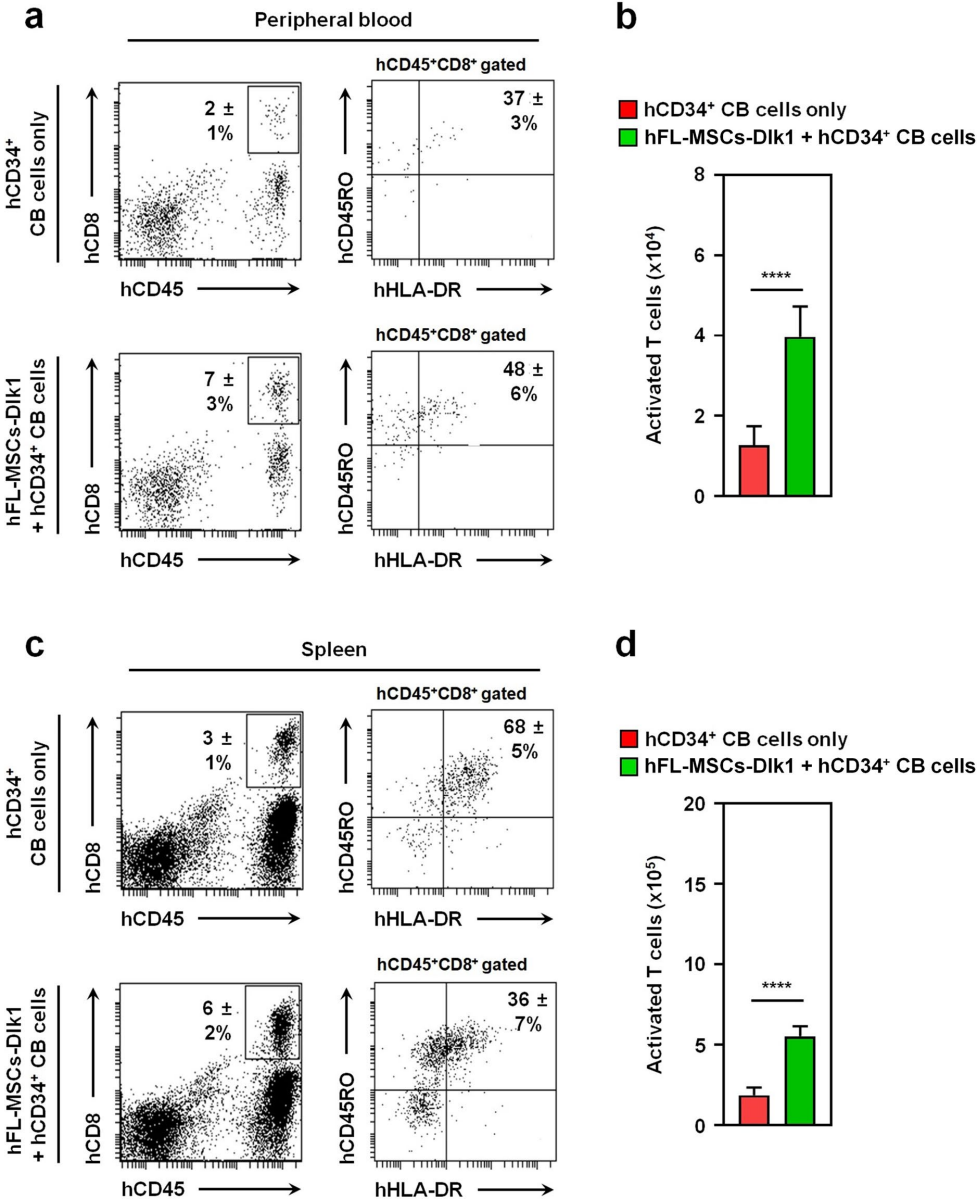
Activated T cells are elevated in humanized NSG mice generated with hDlk1-expressing hFL-MSCs. **(a** and **b)** At 4 weeks after EBV infection, peripheral bloods were isolated from humanized mice generated with hCD34^+^ CB cells alone (*n* = 5) or together with hDlk1-expressing hFL-MSCs (*n* = 5). Cells were prepared as described in Materials and methods and stained with anti-hCD45, hCD3, hCD8, hCD45RO, and hHLA-DR antibodies. Percentages (**a**) and absolute numbers (**b**) of cells were obtained by manual flow cytometric gating and counted. Data are presented as the average of triplicate samples (± S.D.). ****, *p* < 0.0001. (**c** and **d**) At 4 weeks after EBV infection, spleen tissues were isolated from humanized mice generated with hCD34^+^ CB cells alone (*n* = 5) or together with hDlk1-expressing hFL-MSCs (*n* = 5). Cells are prepared, as described in Materials and methods and stained with anti-hCD45, hCD3, hCD8, hCD45RO, and hHLA-DR antibodies. Percentages (**c**) and absolute numbers (**d**) of cells were obtained by manual flow cytometric gating and counted. Data are presented as the average of triplicate samples (± S.D). ****, *p* < 0.0001.

Having shown the above results, we further tried to identify activated T cells, phenotypically presented as hCD45^+^hCD3^+^hCD8^+^hCD45RO^+^hHLA-DR^+^ T cells^41^, in the peripheral blood and spleens of humanized mice challenged with EBV. Percentage and absolute number of activated T cells in the peripheral blood were significantly increased in humanized mice generated using hDlk1-expressing MSCs plus hCD34^+^ CB cells than in humanized mice generated using hCD34^+^ CB cells alone (Fig. 6a, 48 ± 6% *vs.* 37 ± 3%; Fig. 6b, closed green bar *vs.* closed red bar). Although the percentage of activated T cells in spleens were lower in humanized mice generated using hDlk1-expressing MSCs plus hCD34^+^ CB cells than in humanized mice generated using hCD34^+^ CB cells (Fig. 6c, 36 ± 7% *vs.* 68 ± 5%), the absolute number of cells was significantly higher in humanized mice generated using hDlk1-expressing MSCs plus hCD34^+^ CB cells (Fig. 6d, closed green bar *vs.* closed red bar). Altogether, these results suggest that EBV-specific CD8^+^ and activated T cells are effectively generated in humanized mice generated using hDlk1-expressing MSCs plus hCD34^+^ CB cells than in humanized mice generated using hCD34^+^ CB cells alone.

## Discussion

In the present study, we examined in vivo roles of MSCs and Dlk1 in the development and generation of human T cells using humanized NSG mice model established by intrahepatic injection of hCD34^+^ CB cells^8^. We found that human T cell development was severely attenuated in humanized mice co-injected with hFL-MSCs, whereas it was markedly recovered in humanized mice co-injected with hDlk1-expressing hFL-MSCs. After challenge with EBV, interestingly, EBV-specific CD8^+^ T cells were effectively mounted in humanized mice co-injected with hDlk1-expressing hFL-MSCs. More importantly, activated T cells were significantly elevated in humanized mice than in humanized mice generated with hCD34^+^ CB cells alone. These results suggest that Dlk1 can promote human T cell development, thereby functionally enhancing antigen-specific T cell responses in humanized mice.

Human MSCs have been tested as a promising tool for cell-based therapy in both preclinical and clinical trials targeting a variety of human diseases^42,43^. Importantly, the age-related decrease in the frequency and differentiation capacity of adult MSCs affects their cellular functions *in vivo*^44–46^. Therefore, fetal tissue-derived MSCs are being considered as an alternative source to circumvent the diminished potential of adult MSCs^42–44^. Among tissue-resident MSCs, fetal lung- or liver-derived MSCs have shown potential regenerative and immune-modulatory properties as multipotent cells, along with bone marrow (BM)-derived MSCs^47–49^. The fetal lung-derived MSCs represent the unique lung-specific properties and promote the engraftment of umbilical cord blood (UCB)-derived CD34^+^ cells in NOD/SCID mice^20,48^. However, the functional role of MSCs in immune response, especially in T cell response, has been mostly associated with immunosuppressive effects^13–16,23–25^. The inhibitory function of MSCs is achieved by either inhibiting proliferation of T cells or regulating antigen-presentation of DCs^50^. Importantly, it has been reported that fetal liver-derived MSCs produce higher levels of pro-angiogenic, anti-inflammatory, and anti-apoptotic cytokines than those of bone marrow-derived MSCs^50^. Although the detailed analysis for the molecular and cellular mechanisms is required, the immune-modulatory property of MSCs appears to be different depending on their tissue origin. Regarding the in vivo function of MSCs, nevertheless, direct evidence remains unclear. To address the in vivo function of hFL-MSCs, in this study, we utilized humanized NSG mice generated by intrahepatic injection of hCD34^+^ CB cells^8^. Co-injection of hFL-MSCs into humanized mice resulted in marked attenuation of human T cells. Interestingly, hDlk1-expressing hFL-MSCs were markedly detected in the liver and spleen (Supplementary Fig. S5). Moreover, human T cells developed in the humanized mice were detected as $\alpha \beta$ T cells (Supplementary Fig. S6). Considering previous reports showing that fetal liver injected with hCD34^+^ CB cells can effectively provide an environment for T cell development^8^, MSCs in the liver and spleen might be functionally involved in T cell development and proliferation in humanized NSG mice, thereby leading to suppressive effects on T cell development and generation. Our results are consistent with immunomodulatory effects of MSCs reported previously^23–25^.

With these results, we further addressed whether Dlk1 could affect T cell development and generation in humanized mice. It has been well demonstrated that Notch signaling critically regulates cell fate decisions and T cell lineage commitment^31–33^. Moreover, it has been reported that Dlk1 putatively can interact with Notch1, thereby regulating cellular development^34–36^. Therefore, we generated hDlk1-expressing hFL-MSCs and humanized NSG mice injected with hCD34^+^ CB cells plus hDlk1-expressing hFL-MSCs. Interestingly, we found marked increases of human T cells in humanized mice. Moreover, these human T cells were functionally active and proliferated in the presence of hIL-2 (Supplementary Fig. S7). Having shown these results, we further asked whether antigen-specific T cell response could be effectively mounted in humanized mice generated with hCD34^+^ CB cells plus hDlk1-expressing hFL-MSCs. After challenge of EBV, EBV-specific CD8^+^ T cells and hCD45^+^hCD3^+^hCD8^+^hCD45RO^+^hHLA-DR^+^ activated T cells were significantly enhanced in humanized mice. Although the molecular and cellular mechanism by which how Dlk1 is functionally associated with the T cell development and generation could not be addressed in this study, based on previous reports and our current findings, Dlk1 might be able to facilitate T cell development and induce T cell proliferation presumably through Notch signaling, thereby effectively inducing antigen-specific T cell responses in humanized mice.

Based on results from three different experimental sets of humanized NSG mice, we have two conclusions. First, the in vivo function of MSCs in T cell development of humanized NSG mice experimentally established by intrahepatic injection might be functionally associated with immunosuppressive effects. Second, Dlk1 molecule can enhance T cell development and generation in humanized NSG mice, thereby facilitating antigen-specific T cell response. Although the cellular and molecular mechanism setting off the immunosuppressive in vivo effect of MSCs in T cell development and generation of the humanized NSG mice remains unclear, considering therapeutic impacts of MSCs in translational researches, including infectious and regenerative immune diseases, and functional importance of Dlk1-Notch signaling in cell fate decision, our data and humanized NSG mice might contribute to the development of therapeutics targeting various human diseases as a potential animal model system.

## Supplementary Information

Supplementary Information.

## Data Availability

The data that support the findings of this study are available upon request from the corresponding author.
