# Supplementary material for: Human delta like 1-expressing human mesenchymal stromal cells promote human T cell development and antigen-specific response in humanized NOD/SCID/IL-2Rγnull (NSG) mice
Source: Sci Rep. 2021 May 19;11:10603. doi: 10.1038/s41598-021-90110-z (PMC8134586; doi:10.1038/s41598-021-90110-z)
Supplement: Supplementary file 1 — Supplementary Information. [file 41598_2021_90110_MOESM1_ESM.pdf]

Supplementary Information

Human delta like 1-expressing human mesenchymal stromal cells promote human T cell development and antigen-specific response in humanized NOD/SCID/IL-2Rγ^{null} (NSG) mice

Do Hee Kwon[1], Jae Berm Park[2,3], Joo Sang Lee[4,5], Sung Joo Kim[2,6], Bongkum Choi[6,7,8], Ki-Young Lee[1,3,5,8]

[1]Department of Immunology and Samsung Biomedical Research Institute, Sungkyunkwan University School of Medicine, Suwon-Si, Kyonggi-Do 440-746, Korea.

[2]Department of Surgery, Samsung Medical Center, Sungkyunkwan University School of Medicine, Seoul, Republic of Korea.

[3]Department of Health Sciences and Technology, Samsung Advanced Institute for Health Sciences & Technology, Samsung Medical Center, Sungkyunkwan University, Seoul, Republic of Korea.

[4]Department of Precision medicine, Sungkyunkwan University School of Medicine, Suwon-Si, Kyonggi-Do 440-746, Korea.

[5]Single Cell Network Research Center, Sungkyunkwan University School of Medicine, Suwon 16419, Republic of Korea

[6]GenNBio, Inc., Seoul, Republic of Korea.

[7]Department of Medicine, Sungkyunkwan University School of Medicine, Suwon-Si, Kyonggi-Do 440-746, Korea

Supplementary Methods

Flow cytometric analysis

Peripheral blood mononuclear cells (PBMCs) were collected from tail veins of humanized NSG mice at 8, 12, 16, and 20 weeks after transplant. To remove red blood cells (RBCs), cells were treated with 1X RBC Lysis Buffer (Invitrogen) according to the manufacturer's instructions. Single-cell suspensions were prepared from spleen tissues by standard procedures. Cells were collected and washed with 1X PBS. Isolated MNCs were stained with hCD45-allophycocyanin (APC) conjugated, hCD3- peridinin chlorophyll protein-Cyanine5.5 (PerCP-Cy5.5) conjugated, or hCD19-fluorescein isothiocyanate (FITC) conjugated antibody. Flow cytometry analysis was performed using a FACSAria (BD Biosciences). Ten thousand to 1,000,000 events were acquired per sample and analyzed using FACSDiva (BD Biosciences) or FlowJo (BD Biosciences) software. Percentages of cells were obtained by manual flow cytometric gating method. Data are shown as the average of five different mice in each group (± S.D). Thymus, liver, bone marrow, and spleen tissues were isolated from the humanized NSG mice generated with hFL-MSCs-Dlk1 plus $hCD34^{+}$ CB cells at 20 weeks after humanization. Single-cell suspensions were prepared as following standard procedures. Cells were collected, washed with PBS buffer. Incubated with 2ug/ml anti-Dlk1 antibody (abcam, Cambridge, UK) in 1X PBS containing 0.2% BSA and 0.05% sodium azide at 4°C for 30 min, and subsequently incubated cells with the FITC-conjugated goat anti-mouse IgG H&L secondary antibody (abcam) used at a 1:500 dilution in 1X PBS containing 0.2% BSA and 0.05% sodium azide. The cells were incubated in the dark for 30 min at 4°C. Cells were stained with isotype control used mouse IgG2b kappa Isotype Control FITC conjugated antibody (Invitrogen). Percentages of cells were obtained by manual flow cytometric gating method. Data are shown as the average of five different mice in each group (± S.D). Liver tissues were isolated from the humanized NSG mice. Single-cell suspensions were prepared as following standard procedures. Cells were collected and washed with PBS buffer. Mononuclear cells (MNCs) were then isolated by Ficoll-Hypaque density gradient centrifugation. Isolated MNCs were stained with anti-human αβ TCR-FITC or anti-human γδ TCR-phycoerythrin (PE) conjugated antibody. Percentages of cells were obtained by manual flow cytometric gating method. Data are shown as the average of five different mice in each group (± S.D).

Construction of human delta-like 1 (Dlk1) retroviral vector

Human Dlk1 cDNA was synthesized from total RNA extracted from AFT024 cells expressing human Dlk1. cDNA synthesized was performed using 1 μg of purified total RNA in a reaction volume of 20 μl containing 250 mM Tri-HCl, pH 8.3, 375 mM KCl, 15 mM $MgCl_2$, 0.1 M DTT, 10 mM each dNTP, 20 units of RNase inhibitor, 0.5 μg/μl oligo(dT)12-18 primer, and 200 units of SuperScript reverse transcriptase (Invitrogen). RT reaction was performed at 37°C for 1 hr. Synthesized cDNA samples were purified using a QIAquick PCR Purification Kit (Qiagen GmbH) and used as a template for PCR. PCR primers for hDlk1 (accession number: MN003836) were specifically designed as follows: forward, 5’-GGGTCCATGACCGCGACCGAAGCC-3’; reverse, 5’-CCTAGGTTAGATCTCCTCGTCGCC-3’. All amplicons were cloned into BamHI and AvrII sites of a pLXRN retroviral vector (Clontech Laboratories). The pLXRN vector and the purified hDlk1 DNA were linearized with BamHI and AvrII to prepare compatible ends for ligation using T4 DNA ligase (New England Biolabs).

BrdU-labeling assay

Spleen was isolated from humanized mice generated using $hCD34^+$ CB cells together with hDlk1-expressing MSCs. To remove red blood cells (RBCs), cells were treated with 1X RBC Lysis Buffer (Invitrogen) according to the manufacturer's instructions. Single-cell suspensions were prepared. MNCs were then isolated by Ficoll-Hypaque density gradient centrifugation. $hCD3^+$ T cells were sorted using either a MACS human CD3 MicroBead Kit (Miltenyi Biotec, GlodBach, Germany) or an autoMACS™ Cell Separator (Miltenyi Biotec) according to the manufacturer's instructions. For BrdU-labeling assay, we used Bromo-2'-deoxy-uridine Labeling and Detection Kit (Roche) according to the manufacturer's instructions. Briefly, 2×10^5 $hCD3^+$ cells were cultured with 1×10^5 MSCs or Dlk1-expressing MSCs irradiated at 30 Gy in the presence of hIL-2 (20 Unit /ml). At 3 days after culture, cells were stained with BrdU according to the manufacturer's instructions. Cells were stained with anti-hCD3- PerCP-Cy5.5 or anti-BrdU-FITC conjugated antibody. Flow cytometry analysis was performed on a FACSAria (BD Biosciences). Ten thousand to 1,000,000 events were acquired per sample and analyzed using FACSDiva (BD Biosciences) or FlowJo (BD Biosciences) software.

Percentages of cells were obtained by manual flow cytometric gating method. Data are shown as the average of three different experimental samples (± S.D).

Supplementary Results

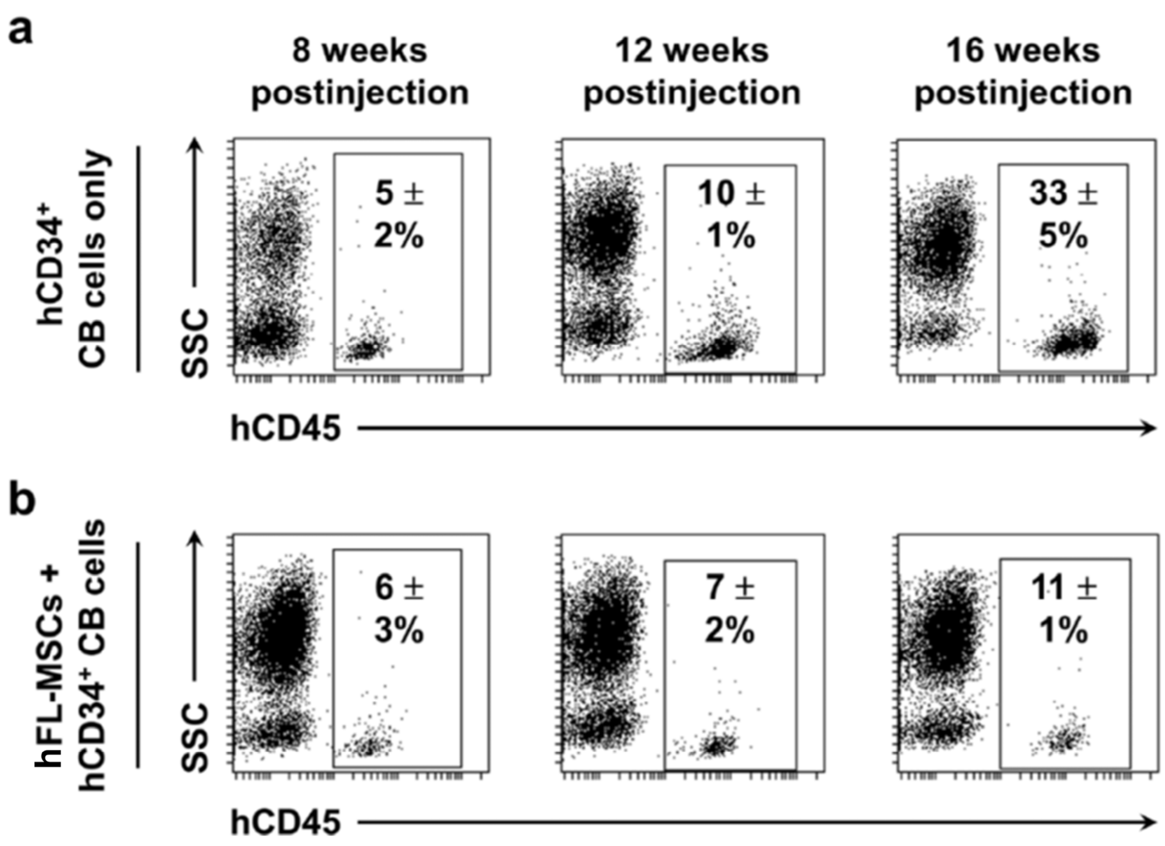

Figure S1 Reconstitution of hCD45^{+} cells in the humanized mice. (**a** and **b**) Peripheral blood mononuclear cells (PBMCs) were collected from tail veins of humanized NSG mice (**a**, hCD34^{+} CB cell alone; **b**, hFL-MSCs plus hCD34^{+} CB cells) at 8, 12, and 16 weeks after transplant, and stained with anti-hCD45 antibody, as described in the Supplementary Materials and Methods. Percentages of hCD45^{+} cells were obtained by manual flow cytometric gating method. Data are shown as the average of five different mice in each group (± S.D).

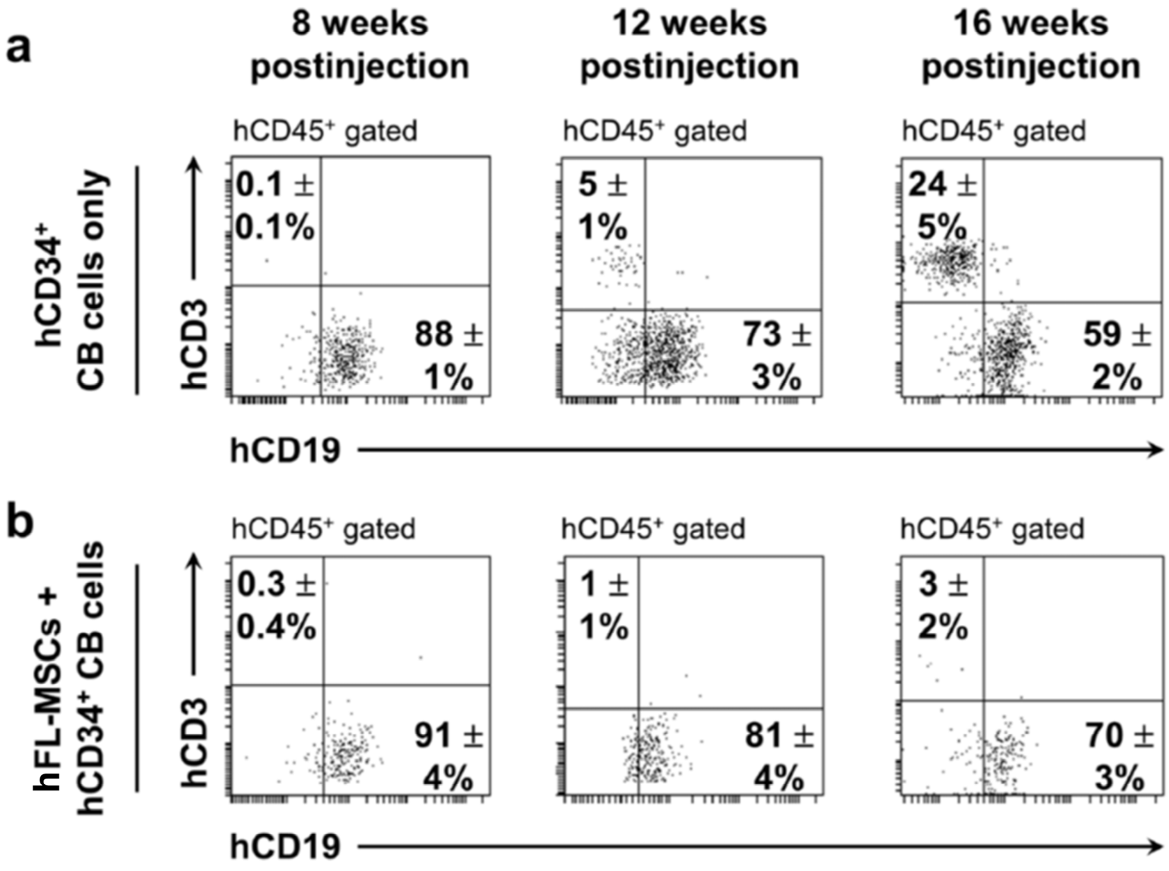

Figure S2 Reconstitution of hCD45⁺hCD3⁺ cells in the humanized mice. (**a** and **b**) Peripheral blood mononuclear cells (PBMCs) were collected from tail veins of humanized NSG mice (**a**, hCD34⁺ CB cell alone; **b**, hFL-MSCs plus hCD34⁺ CB cells) at 8, 12, and 16 weeks after transplant, and stained with anti-hCD45 and anti-hCD3 antibodies, as described in the Supplementary Materials and Methods. Percentages of hCD45⁺hCD3⁺ cells were obtained by manual flow cytometric gating method. Data are shown as the average of five different mice in each group (± S.D).

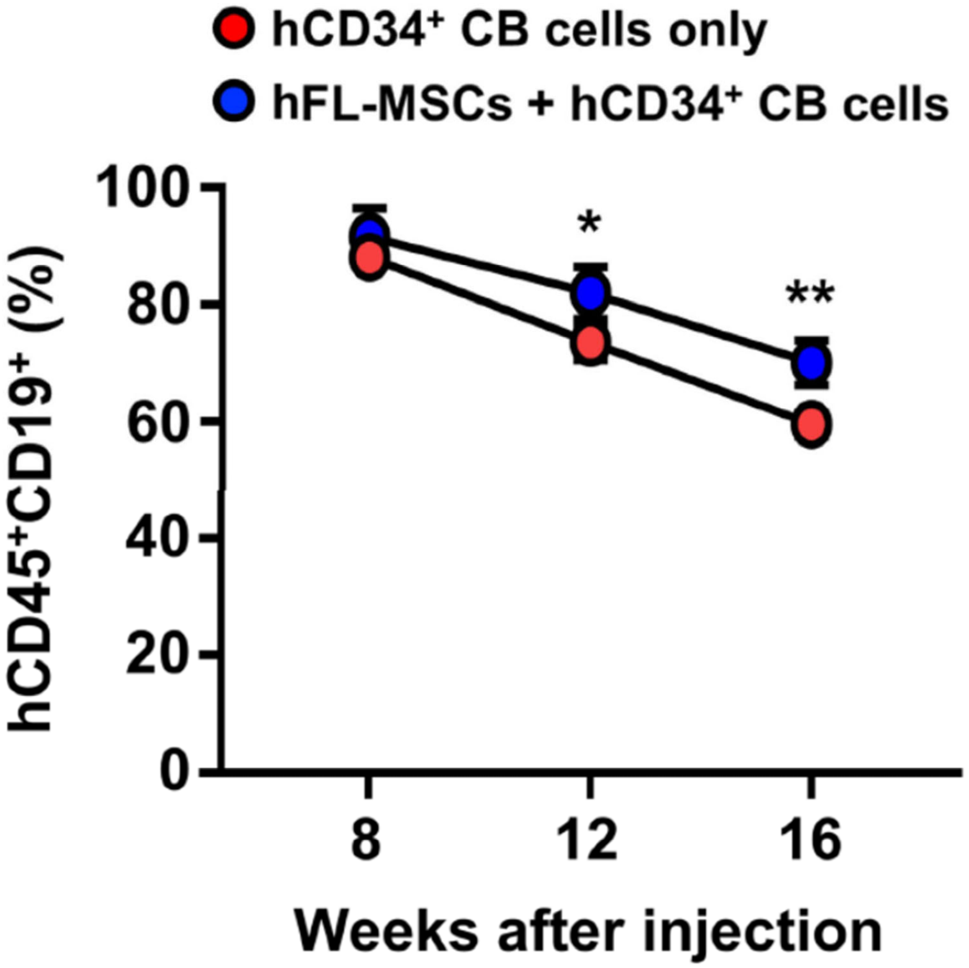

Figure S3 Reconstitution of $hCD45^+hCD19^+$ cells in the humanized mice. Peripheral blood mononuclear cells (PBMCs) were collected from tail veins of humanized NSG mice at 8, 12, and 16 weeks after transplant, and stained with anti-hCD45 and anti-hCD19 antibodies, as described in the Supplementary Materials and Methods. Percentages of $hCD45^+hCD19^+$cells were obtained by manual flow cytometric gating method. Data are shown as the average of five different mice in each group (± S.D). *, $p < 0.05$; **, $p < 0.01$.

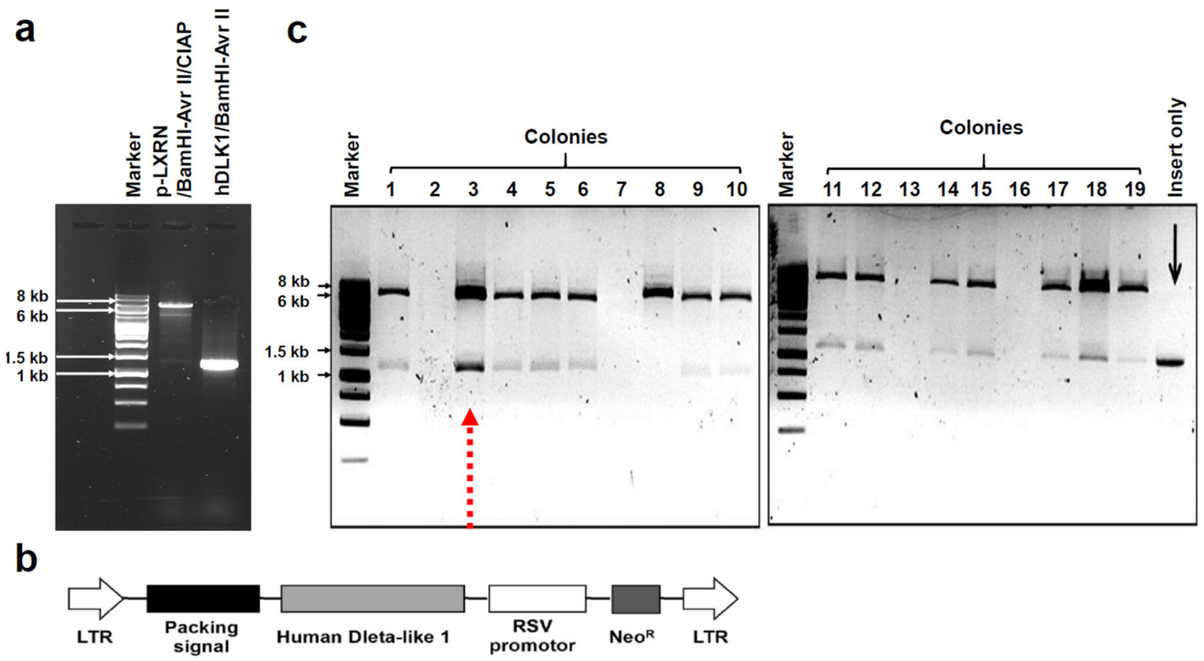

Figure S4 Construction of retroviral vector containing human delta-like 1 (Dlk1) gene. A Prepared the pLXRN retroviral vector and insert gene, hDlk1, and clones were performed restriction enzyme analysis and run on 1% agarose gel. A 1 kb ladder (BioLabs) is used to mark band sizes. Gels were photographed using Gel Doc XR+ Gel Documentation System (Bio-Rad). (**a**) Purified pLXRN retroviral vector (6.4 kb) and amplified hDlk1 gene (1.2 kb) were digested with BamHI and AvrII. (**b**) Schematic representation of pLXRN retroviral vectors that contains hDlk1 gene. (**c**) After cloning, selected clones were performed restriction enzyme digestion by BamHI and AvrII and the results confirmed recombinant pLXRN vector and contained the desired gene. Load hDlk1/BamHI-AvrII as a control and compared band size with digested hDlk1 gene from pLXRN-Dlk1 plasmid DNA. #3 colony was used in this study (Red arrow).

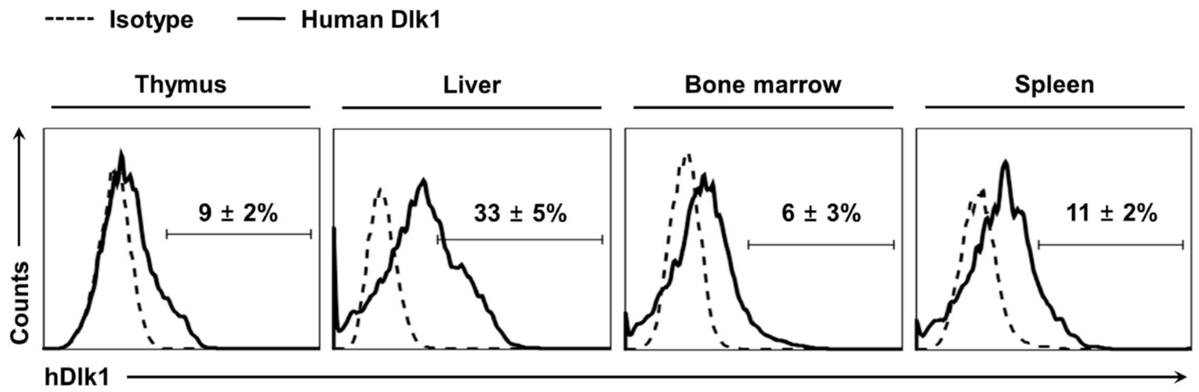

Figure S5 Identification of hFL-Dlk1 MSCs in the humanized mice. Thymus, liver, bone marrow, and spleen tissues were isolated from the humanized NSG mice generated with hFL-MSCs-Dlk1 plus hCD34^{+} CB cells. Single-cell suspensions were prepared as following standard procedures. Cells were collected, washed with PBS buffer, and were stained with isotype control (mouse IgG2b kappa) or anti-Dlk1 primary and FITC-conjugated goat anti-mouse IgG H&L secondary antibody. Percentages of cells were obtained by manual flow cytometric gating method. Data are shown as the average of five different mice in each group (± S.D)

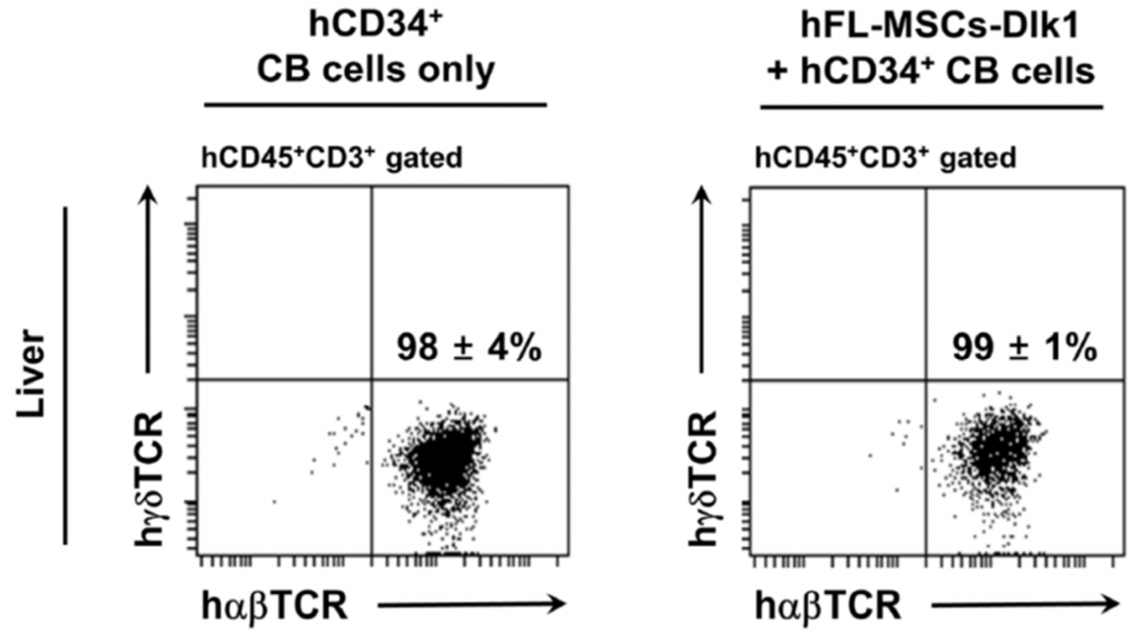

Figure S6. Analysis of αβ TCR or γδ TCR human T cells in the humanized mice. Liver tissues were isolated from the humanized NSG mice generated hCD34$^+$ CB cells only or FL-MSC-Dlk1 plus hCD34$^+$ CB cells. Single-cell suspensions were prepared as following standard procedures. Cells were collected and washed with 1X PBS. Mononuclear cells (MNCs) were then isolated by Ficoll-Hypaque density gradient centrifugation. Isolated MNCs were stained with anti-human αβ TCR-FITC or anti-human γδ TCR-PE conjugated antibody. Percentages of cells were obtained by manual flow cytometric gating method. Data are shown as the average of five different mice in each group (± S.D).

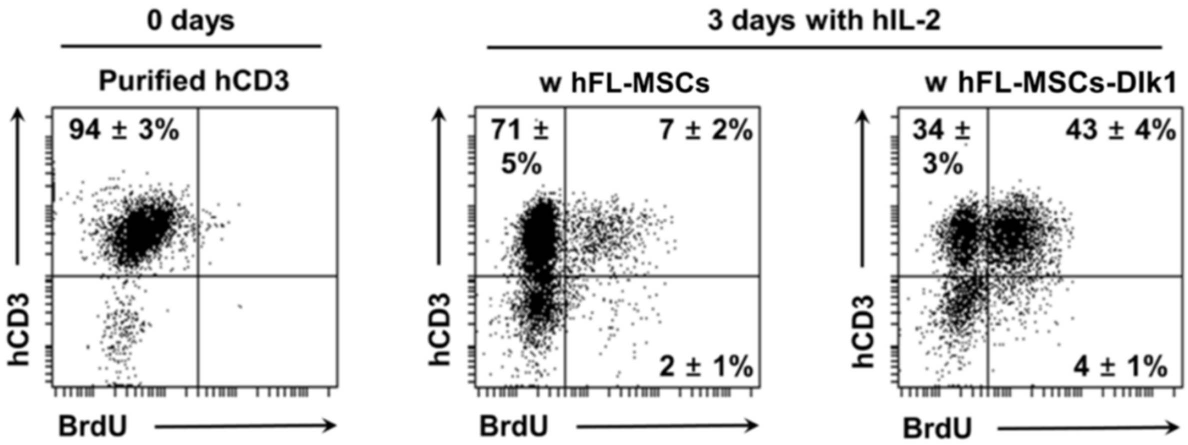

Figure S7. BrdU-labeling assay for cell proliferation analysis. Spleen was isolated from humanized mice generated using hCD34$^+$ CB cells together with hFL-MSCs or hDlk1-expressing hFL-MSCs. hCD3$^+$ T cells were sorted, and BrdU-labeling assay was performed, as described in the Supplementary Materials and Methods. Percentages of cells were obtained by manual flow cytometric gating method. Data are shown as the average of three different experimental samples (± S.D).
